# Inter-brain coupling reflects disciplinary differences in real-world classroom learning

**DOI:** 10.1038/s41539-023-00162-1

**Published:** 2023-05-02

**Authors:** Jingjing Chen, Penghao Qian, Xinqiao Gao, Baosong Li, Yu Zhang, Dan Zhang

**Affiliations:** 1 Department of Psychology, School of Social Sciences, Tsinghua University, Beijing, China; 2 Tsinghua Laboratory of Brain and Intelligence, Tsinghua University, Beijing, China; 3 College of Information and Electrical Engineering, China Agricultural University, Beijing, China; 4 Beijing No. 19 High School, Beijing, China; 5 College of Education, Zhejiang Normal University, Jinhua, China; 6 Institution of Education, Tsinghua University, Beijing, China

**Keywords:** Social neuroscience, Human behaviour

## Abstract

The classroom is the primary site for learning. A vital feature of classroom learning is the division of educational content into various disciplines. While disciplinary differences could substantially influence the learning process toward success, little is known about the neural mechanism underlying successful disciplinary learning. In the present study, wearable EEG devices were used to record a group of high school students during their classes of a soft (Chinese) and a hard (Math) discipline throughout one semester. Inter-brain coupling analysis was conducted to characterize students’ classroom learning process. The students with higher scores in the Math final exam were found to have stronger inter-brain couplings to the class (i.e., all the other classmates), whereas the students with higher scores in Chinese were found to have stronger inter-brain couplings to the top students in the class. These differences in inter-brain couplings were also reflected in distinct dominant frequencies for the two disciplines. Our results illustrate disciplinary differences in the classroom learning from an inter-brain perspective, suggesting that an individual’s inter-brain coupling to the class and to the top students could serve as potential neural correlates for successful learning in hard and soft disciplines correspondingly.

## Introduction

Classroom learning, where many students learn together under the guidance of a teacher in a classroom, is the primary site for formal learning. Due to its practical importance for personal development, classroom learning has drawn consistent attention from the fields of education and psychology^1–3^. It has also been considered an ideal starting point for real-world neuroscience for its semi-controlled structures^4^.

A vital feature of classroom learning is the division of educational content into various disciplines (e.g., Math, history, physics, or language courses). It is widely acknowledged that disciplinary differences could substantially influence classroom learning. The “hard-soft” dimension is possibly one of the most influential frameworks regarding disciplinary differences^5^. Hard disciplines (e.g., math, natural science, and engineering) are known for their relatively hierarchical, linear knowledge structure and straightforward, uncontentious learning contents. Soft disciplines are usually associated with loose-structured, non-linear knowledge and contents that require more constructive and interpretative activity (e.g., history, philosophy, and language courses)^5–7^. Differences in disciplinary knowledge between hard and soft disciplines have been shown to influence teachers’ teaching objectives, which, in turn, influences what the most optimal disciplinary learning process is for students^8,9^. For instance, it has been proposed that students prioritize attending to knowledge from teachers when learning hard disciplines over their soft counterparts^10^. Nonetheless, it should be noted that disciplinary differences in the successful learning process have mainly been inferred based on indirect data such as expert evaluation, retrospective self-reports, and learning outcomes^7,10,11^. There is a dearth of empirical studies directly addressing the learning process itself^12^.

The recently-developed inter-brain coupling analysis has demonstrated its potential as a powerful tool to directly assess the learning process. Unlike traditional neuroscience methods which identify brain activities of interest in reference to a parametric task condition (or contrasts between conditions), the inter-brain coupling approach identifies brain activities of interest in reference to the brain activities of others in the same task condition^13^. Inter-brain coupling analysis was proposed based on the findings that similar brain activities emerged across participants in the same naturalistic task (e.g., watching a movie or listening to a story)^14,15^. It has been argued that similar brain activities across participants could reflect the cognitive processing related to the shared tasks, from low-level sensory processing to high-level semantic or emotional processing^16^. Therefore, by computing an individual’s inter-brain coupling (i.e., similarity) to other people who share the same tasks, the inter-brain coupling approach can circumvent the need for an explicit model of tasks while still tracking the task-related responses^13^. This characteristic of inter-brain coupling makes it ideal for studying the classroom learning process where a parametric task design is difficult or even impossible to achieve due to the complexity of the real-world classroom.

Recent studies have shown that inter-brain coupling analysis is able to investigate the learning process during typical learning scenarios such as attending lectures, watching videos, or group discussions^4,17,18^. More importantly, these studies have highlighted the importance of “student-class coupling” (i.e., the inter-brain coupling between one student and all their other classmates). Previous studies have suggested that shared brain responses across classmates reflect the characteristics of shared external stimuli in the classroom (i.e., courses given by teachers or videos)^4,19^. Therefore, by computing the similarity between brain activities of one student and that of all their other classmates, student-class coupling is expected to reflect the degree to which students attend the course content^4,18,19^, thus characterizing students’ learning process. Indeed, student-class coupling during the learning process was found to positively correlate with students’ engagement^4^, memory retention performance^19^, and final exam scores^18^.

Moreover, it should be noted that the courses investigated in these inter-brain studies to date belong to hard disciplines, such as biology, computer science, and physics. Learning hard disciplines requires the mastery of course content (e.g., the application of fixed mathematical rules)^7^. Since student-class coupling could reflect students’ engagement in the course content, strong student-class coupling during classroom learning is expected to lead to good learning outcomes in hard disciplines^4,18^. Differences in learning goals for soft disciplines may, however, result in differences in the most optimal learning process for students to meet the expectations of their teachers. For example, soft disciplines value personal construction and creativity compared to hard disciplines^7^. Students’ engagement in the course content (as reflected by student-class coupling) may not necessarily associate with learning outcomes in soft disciplines, which might undermine the importance of student-class coupling.

Student-expert coupling could be an alternative candidate to evaluate whether a student’s learning process is optimal in soft disciplines. In educational practice, an expert‐like mastery of knowledge has been regarded as the target for students’ learning and has been linked to good learning outcomes^20^. For example, a recent functional magnetic resonance imaging (fMRI) study recorded the neural activities of both students and experts during the recap task and the final exam in a computer science course. With inter-brain coupling analysis, students’ neural alignment (coupling) to the experts were found to positively predict their final exam scores^18^. While experts have been regarded as a well-established exemplar for successful learning, top students (students in a class with top learning outcomes) could also serve as a possible reference for successful learning since good learning outcomes have long been associated with an effective learning process^21^. Moreover, since experts may learn qualitatively differently due to a broader background knowledge compared with novices^18^, the top-performing students with similar prior knowledge about the to-be-learned content may be particularly effective as an exemplar for the learning process starting as a novice. Therefore, it may be conceivable to take student-top coupling, i.e., the average inter-brain coupling from one student to their top-performing peers of all the classmates during the learning process, as a neural correlate to evaluate students’ learning process in soft disciplines. However, no inter-brain study has addressed the issue.

The real-world classroom is expected to serve as an ideal site to investigate the disciplinary differences in successful learning. Compared to the conventional laboratory-based studies that have mainly focused on strictly-controlled, parametric experimental designs (i.e., based on contrasts across simplified learning tasks to isolate targeted factors in disciplinary differences) remotely resembling real-world learning^13,22,23^, classroom-based studies have an advantage due to their high ecological validity since they can directly reflect the complex and dynamic disciplinary learning process that occurs on a daily basis. The recent development of wearable electroencephalogram (EEG) devices has enabled researchers to track students’ learning processes in real-classroom settings^24–27^. For instance, EEG devices in the form of a headband could support the easy acquisition of EEG data from an entire class of students on account of their portability, usability, low purchase, and running costs. Wearable EEG devices have been proven to be effective in tracking students’ sustained attention, situational interests, and engagement during their classroom learning processes^4,28,29^. The ecologically naturalistic paradigm with wearable neuroimaging technologies is expected to provide insights into understanding the disciplinary differences “in the wild” and offer a possible “fast lane” to apply neuroscience findings into educational theoretical construction and practical application^25,30^.

**Fig. 1:**
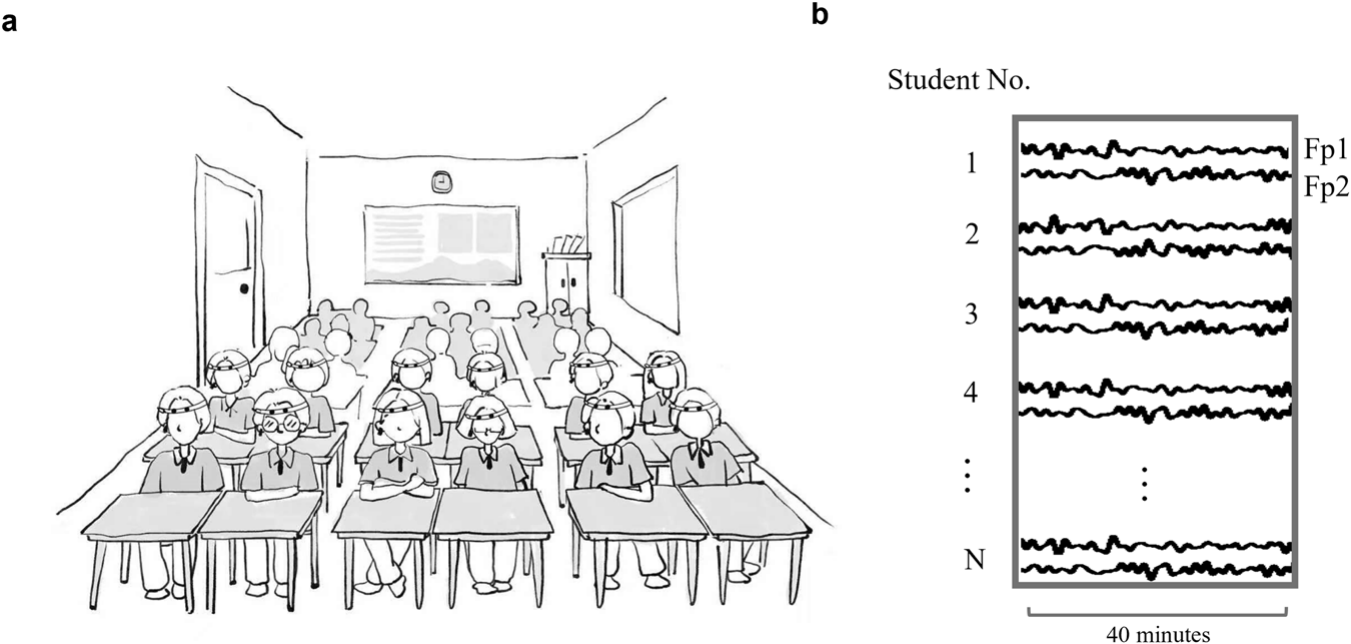
Experiment paradigm. **a** An illustration of the experimental setup of students wearing an EEG headband on their forehead with a reference electrode at the right ear lobe during their regular classroom learning; **b** an illustration of the recorded EEG signal during a session; EEGs were recorded at Fp1 and Fp2 for all the students for 40 min during a session.

Therefore, the present study investigated disciplinary differences in the successful classroom learning process. A class of students from grade 10 from a high school in Beijing volunteered to join the study. Wearable EEG headbands with two dry electrodes covering Fp1/2 were chosen to record students’ brain signals during their regular Math and Chinese sessions in the classroom throughout one semester (as demonstrated in Fig. 1). Math and Chinese (the native language in China) were chosen as representative courses for the hard discipline and soft discipline, respectively, as they are two of the most important compulsory courses before college in China.

**Fig. 2:**
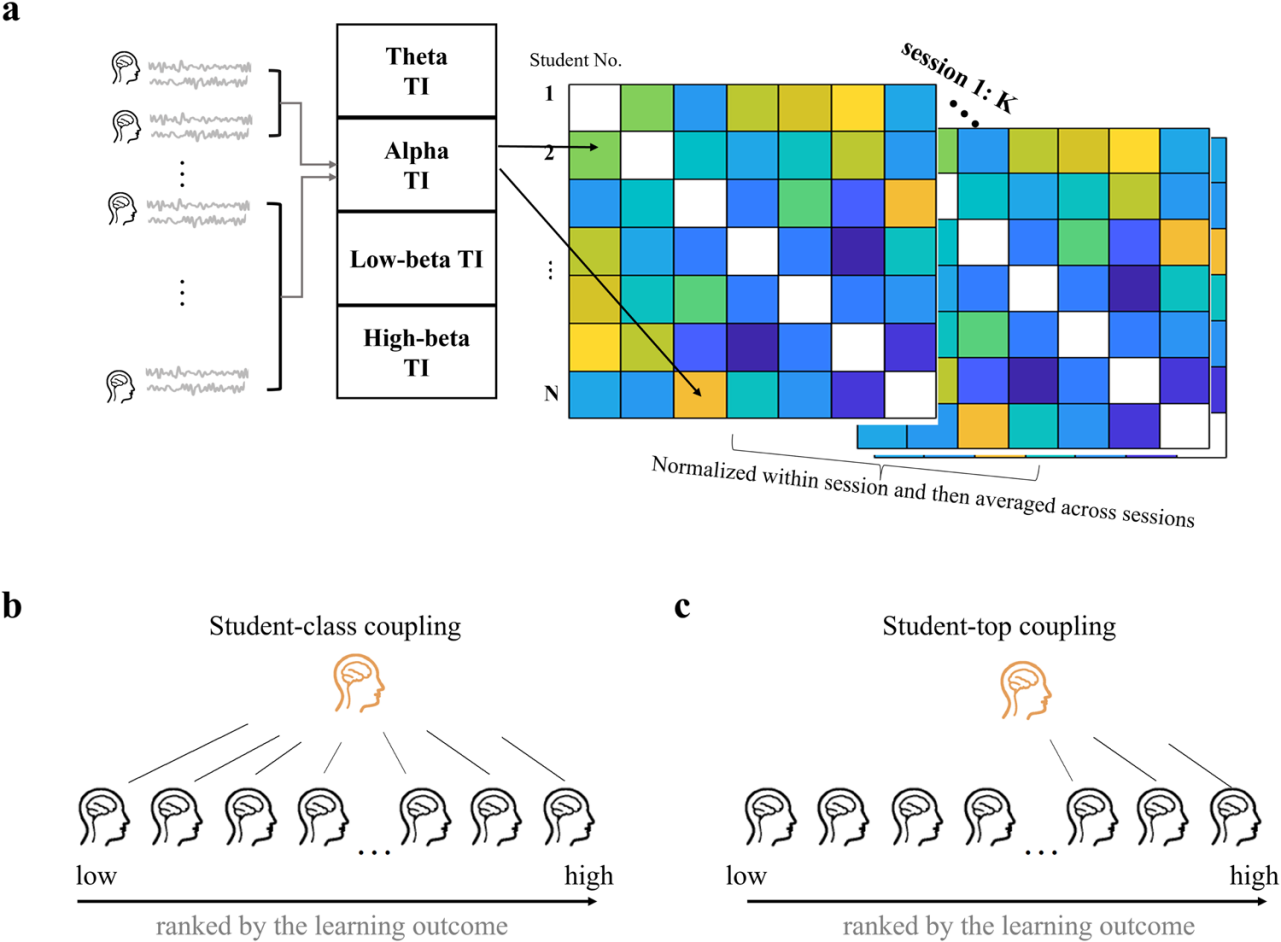
A schematic illustration of the inter-brain coupling analysis. **a** Computations of pairwise total interdependence (TI) matrix for each pair of students for each session, at the frequency bands of theta, alpha, low-beta, and high-beta. The TI values were then normalized within each session and averaged across sessions to obtain an inter-brain coupling value for each pair of students. **b** Student-class coupling was obtained by averaging TI values over all possible pairwise combinations between one student and the rest of the class. **c** Student-top coupling was computed by averaging TI values over all possible pairwise combinations between one student and all the top students in the class, except for this student himself/herself if the student was one of the top students. Here, *N* is the number of students, *K* is the total number of sessions.

As shown in Fig. 2, inter-brain coupling analyses were conducted to assess students’ classroom learning process using the total interdependence (TI) method^4,17,31^. Specifically, student-class coupling (the inter-brain coupling between one student and all their other classmates) and student-top coupling (the inter-brain coupling between one student and their top peers) were computed, and the associations between student-class/top coupling and the learning outcomes for Math/Chinese courses were explored. Since student-class coupling has been reported to be positively correlated with the learning outcomes in different hard disciplines (e.g., biology, computer science, and chemistry) and has been proposed to reflect the students’ engagement in the course content^4,18,19^, we hypothesized that student-class coupling could be a potential neural correlate to characterize the optimal learning process during Math courses as a typical hard discipline. Accordingly, student-class coupling was expected to be associated with the learning outcomes for Math courses. For Chinese as a typical soft discipline, however, due to the differences in learning requirements with its hard counterpart^7^, the engagement in the course content (as reflected by student-class coupling) may not be sufficient to support a good learning outcome. Rather, the personal construction and interpretation of top students could be a better reference to reflect an effective Chinese learning process^7,21^. Hereby, it is plausible to consider student-top coupling as a promising candidate to be associated with the learning outcomes for Chinese courses. As soft disciplines have not been sufficiently addressed in previous inter-brain studies and student-top coupling has not been previously studied, the hypothesis regarding Chinese learning and student-top coupling is exploratory in nature. In particular, the student-top couplings were computed by varying the number of top students to evaluate the reliability of possible findings. The students’ final exam scores for Chinese and Math were taken to indicate their learning outcomes at the end of this semester. Pearson’s correlation analysis between inter-brain patterns and learning outcomes was conducted to identify the inter-brain correlates of successful classroom learning, with nonparametric permutation tests further verifying the correlation (see more details in the “Nonparametric permutation tests” section). Correlations were regarded as significant only if both Pearson’s *p* and permutation *p* were smaller than 0.05. No clear hypothesis regarding the specifically involved frequency band was formulated due to limited evidence. Therefore, four frequency bands: theta (4–8 Hz), alpha (8–13 Hz), low-beta (13–18 Hz), and high-beta (18–30 Hz) were analyzed. Any discovery would advance our understanding of the neural mechanism behind the successful disciplinary learning process in the classroom.

## Results

### Theta-band student-class coupling reflects successful classroom learning for Math

**Fig. 3:**
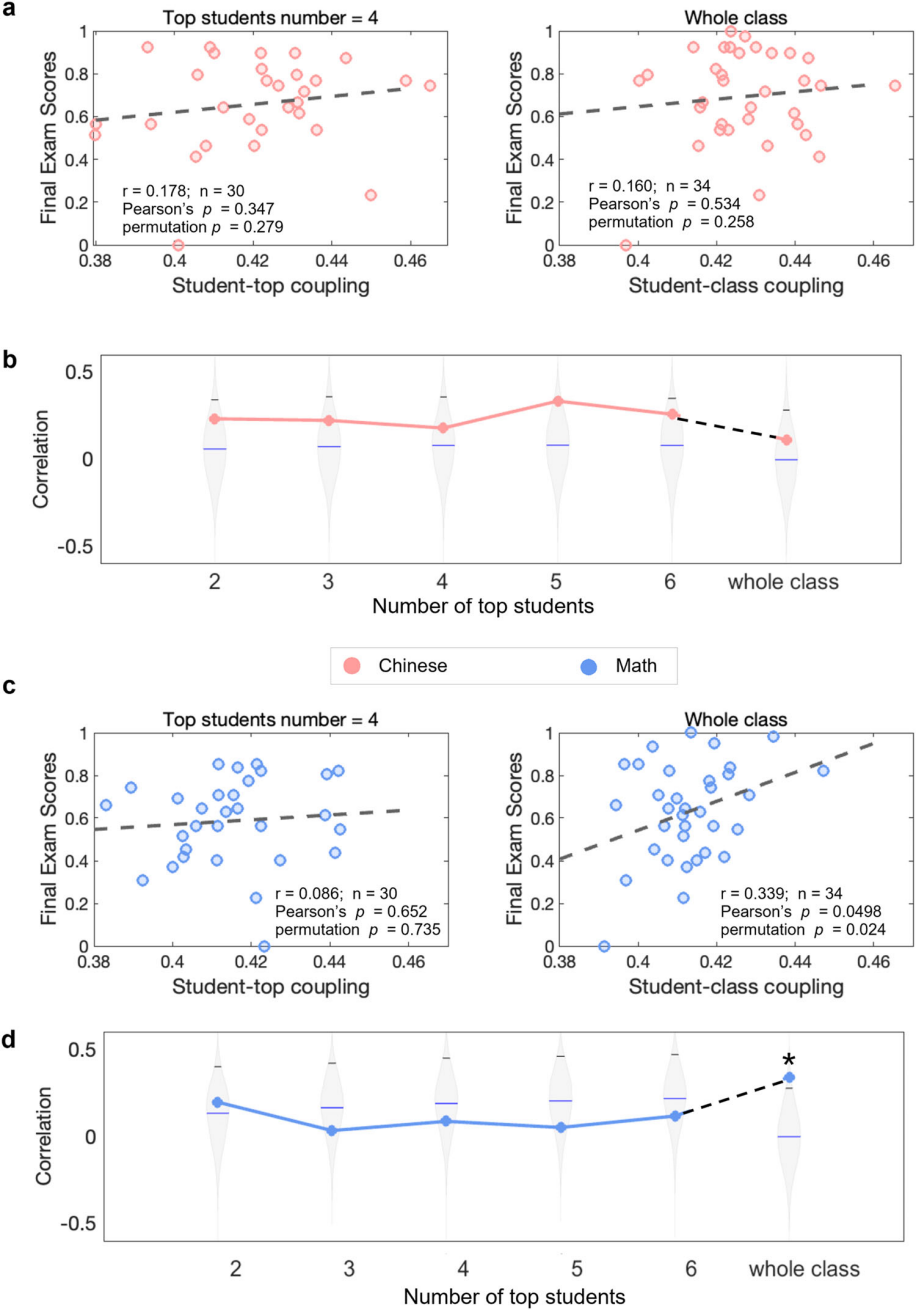
Correlations between theta-band student-top/class coupling and an individual’s final exam score for Chinese and Math. **a** Scatter plots between theta-band student-top couplings (left, top students’ number = 4) and theta-band student-class couplings (right) and the final exam scores of Math. **b** Correlation *r* values as a function of the number of top students included in the calculation of student-top couplings. **c** Scatter plots between theta-band student-top couplings (left, top students’ number = 4) and theta-band student-class couplings (right) and the final exam scores of Chinese. **d** Correlation *r* values as a function of the number of top students included in the calculation of student-top couplings. The violin plots showed the distribution of correlation *r* values generated by the 5000 shuffled versions, the black line indicated the 95th percentile of the distribution, and the blue line indicated the mean value of the distribution. The star indicates a significant correlation when both Pearson’s *p* and permutation *p* were smaller than 0.05. The colored lines in (**b**) and (**d**) represent the trend of how correlation values changed with the number of top students. The color was chosen according to the corresponding discipline (pink for Chinese and blue for Math). Note that the top students themselves were not included in the correlation analysis.

Theta-band student-class couplings during Math sessions were found to be positively correlated with the final exam scores for Math (Fig. 3c, d), suggesting theta-band student-class coupling could serve as a neural correlate of successful disciplinary learning in Math. The students with better learning outcomes in Math were found to have stronger inter-brain couplings to other classmates (*r* = 0.339, Pearson’s *p* = 0.0498, uncorrected for multiple comparisons, same below, *n* = 34). The permutation test also showed that the correlation between theta-band student-class couplings and original Math learning outcomes was significantly higher than the ones generated by the 5000 shuffled versions (see the distribution in Fig. 3d, permutation *p* = 0.024, uncorrected for multiple comparisons, same below). All the permutation *p* values are listed in Supplementary Table S1.

No significant correlations between theta-band student-top couplings and Math scores were found, with the number of top students included in the calculation of student-top couplings varying from 2 up to 6. Additionally, no significant correlations were observed between theta-band inter-brain couplings and the final exam scores for Chinese, neither in student-top couplings nor in student-class couplings (Fig. 3a, b; student-top coupling: *r* = 0.178, Pearson’s *p* = 0.347, permutation *p* = 0.279, *n* = 30, top students’ number = 4; student-class coupling: *r* = 0.110, Pearson’s *p* = 0.534, permutation *p* = 0.258, *n* = 34).

### Alpha-band student-top coupling reflects successful classroom learning for Chinese

**Fig. 4:**
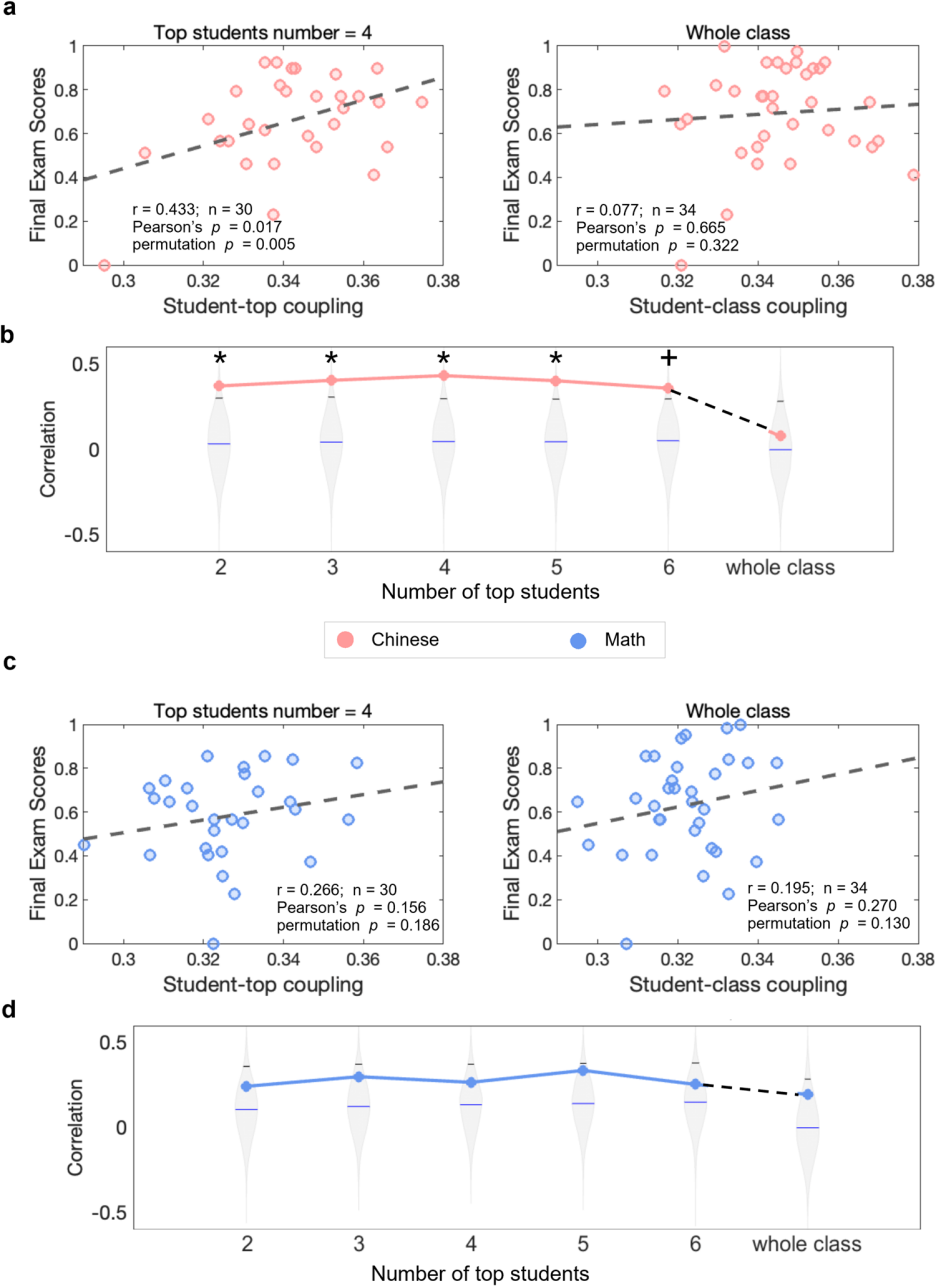
Correlations between alpha-band student-top/class coupling and an individual’s final exam score of Chinese and Math. **a** Scatter plots between alpha-band student-top coupling (left, top students’ number = 4) and alpha-band student-class coupling (right) and the final exam score of Chinese. **b** Correlation *r* values as a function of the number of top students included in the calculation of student-top couplings. **c** Scatter plots between alpha-band student-top couplings (left, top students’ number = 4) and alpha-band student-class couplings (right) and the final exam scores of Math. **d** Correlation *r* values as a function of the number of top students included in the calculation of student-top couplings. The violin plots showed the distribution of correlation *r* values generated by the 5000 shuffled versions, the black line indicated the 95th percentile of the distribution, and the blue line indicated the mean value of the distribution. The star indicates a significant correlation when both Pearson’s *p* and permutation *p* were smaller than 0.05. The cross indicates a marginal significance. The colored lines in (**b**) and (**d**) represent the trend of how correlation values changed with the number of top students. The color was chosen according to the corresponding discipline (pink for Chinese and blue for Math). Note that the top students themselves were not included in the correlation analysis.

Alpha-band student-top coupling during Chinese sessions was significantly correlated with the final exam scores for Chinese, suggesting alpha-band student-top coupling could serve as the neural correlate of successful disciplinary learning in Chinese (Fig. 4a). The students with better learning outcomes in Chinese were found to have stronger inter-brain couplings to the top-performing students (*r* = 0.433, Pearson’s *p* = 0.017, *n* = 30, top students’ number = 4). The permutation showed that the correlation between original alpha-band student-top couplings and Chinese learning outcomes was significantly higher than the ones generated by the 5000 shuffled versions (permutation *p* = 0.005; Fig. 4b). The correlation remained significant or marginally significant when the number of top students included in the calculation varied from 2 to 6 (Pearson’s *p* values range from 0.017 to 0.061; permutation *p* values range from 0.005 to 0.020).

No significant correlations between alpha-band student-class couplings and the final exam scores of Chinese were found (*r* = 0.077, Pearson’s *p* = 0.665, permutation *p* = 0.322, *n* = 34). Moreover, no significant correlations were observed between alpha-band inter-brain couplings and the final exam scores of Math, neither in student-top couplings nor in student-class couplings (Fig. 4c, d; student-top coupling: *r* = 0.266, Pearson’s *p* = 0.156, permutation *p* = 0.186, *n* = 30, top students’ number = 4; student-class coupling: *r* = 0.195, Pearson’s *p* = 0.270, permutation *p* = 0.130, *n* = 34).

### Frequency-specificity of outcome-related inter-brain coupling

**Fig. 5:**
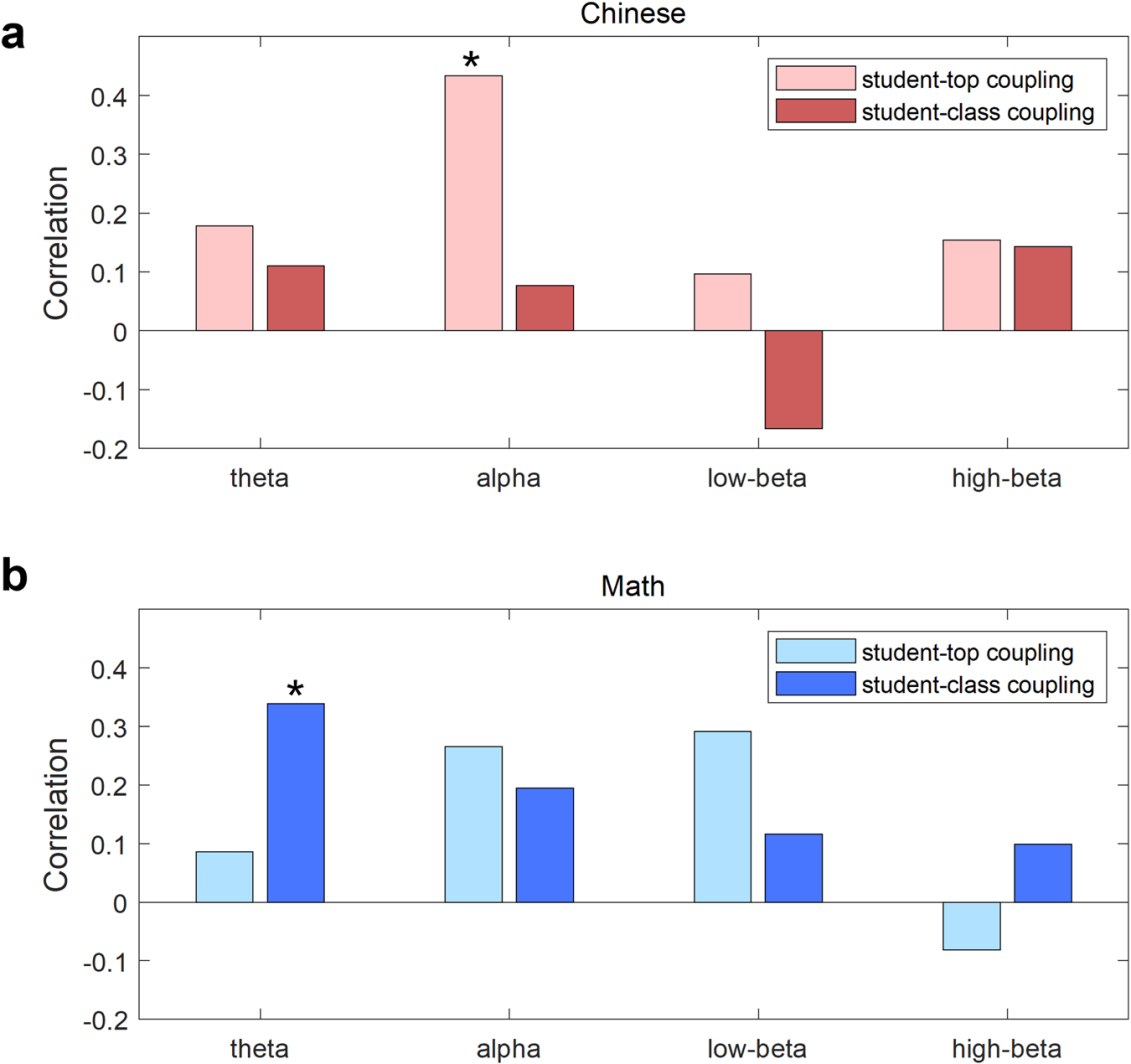
The summary of correlations between inter-brain couplings and learning outcomes. Correlation *r* values between inter-brain couplings and the final exam scores at the theta, alpha, low-beta, and high-beta bands for (**a**) Chinese and (**b**) Math. Bars with a lighter color indicated student-top-coupling-based correlations (top students’ number = 4), and bars with a darker color indicated student-class-coupling-based correlations. Stars indicated a significant correlation when both Pearson’s *p* and permutation *p* were smaller than 0.05.

The correlation results between students’ inter-brain couplings and their learning outcomes in the four frequency bands (theta, alpha, low-beta, and high-beta) were summarized in Fig. 5 to demonstrate the frequency-specificity of outcome-related inter-brain couplings. Inter-brain coupling in the theta and alpha bands was found to correlate with the final exam scores (as shown above) significantly. In contrast, the inter-brain coupling at the low-beta and high-beta bands failed to reach significance.

**Fig. 6:**
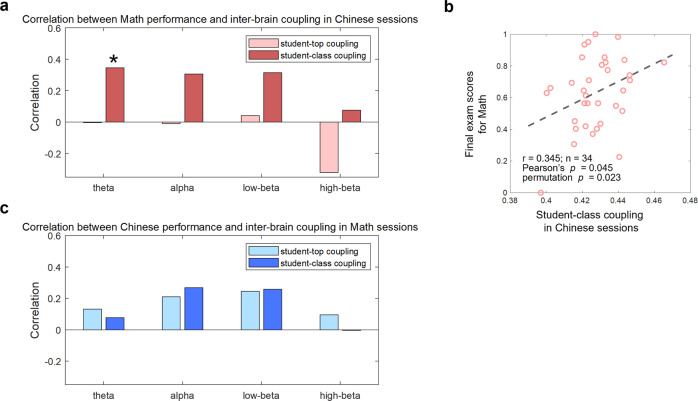
The discipline-specific analysis results. The correlations were re-calculated by computing the correlation between inter-brain couplings during Chinese sessions and the final exam scores of Math (**a**) and vice versa (**c**). **b** Scatter plots between theta-band student-class coupling during Chinese sessions and the final exam scores of Math. Bars with a lighter color indicated student-top-coupling-based correlations (top students’ number = 4), and bars with a darker color indicated student-class-coupling-based correlations. The stars indicated a significant correlation when both Pearson’s *p* and permutation *p* were smaller than 0.05.

With the observed positive correlations between the inter-brain couplings and the corresponding discipline-specific learning outcomes, a further exploratory analysis was conducted to test whether these observations are discipline-specific. The correlations were re-calculated by computing the correlation between inter-brain couplings during Math sessions and the final exam scores of Chinese and vice versa. Details of the analysis can be found in the Methods sections. As shown in Fig. 6b, theta-band student-class couplings during Chinese sessions were significantly correlated with the final exam scores of Math (student-class coupling: *r* = 0.345, Pearson’s *p* = 0.045, permutation *p* = 0.023, *n* = 34). No other correlations reached a significant level.

### Single-brain features fail to reflect successful learning

**Fig. 7:**
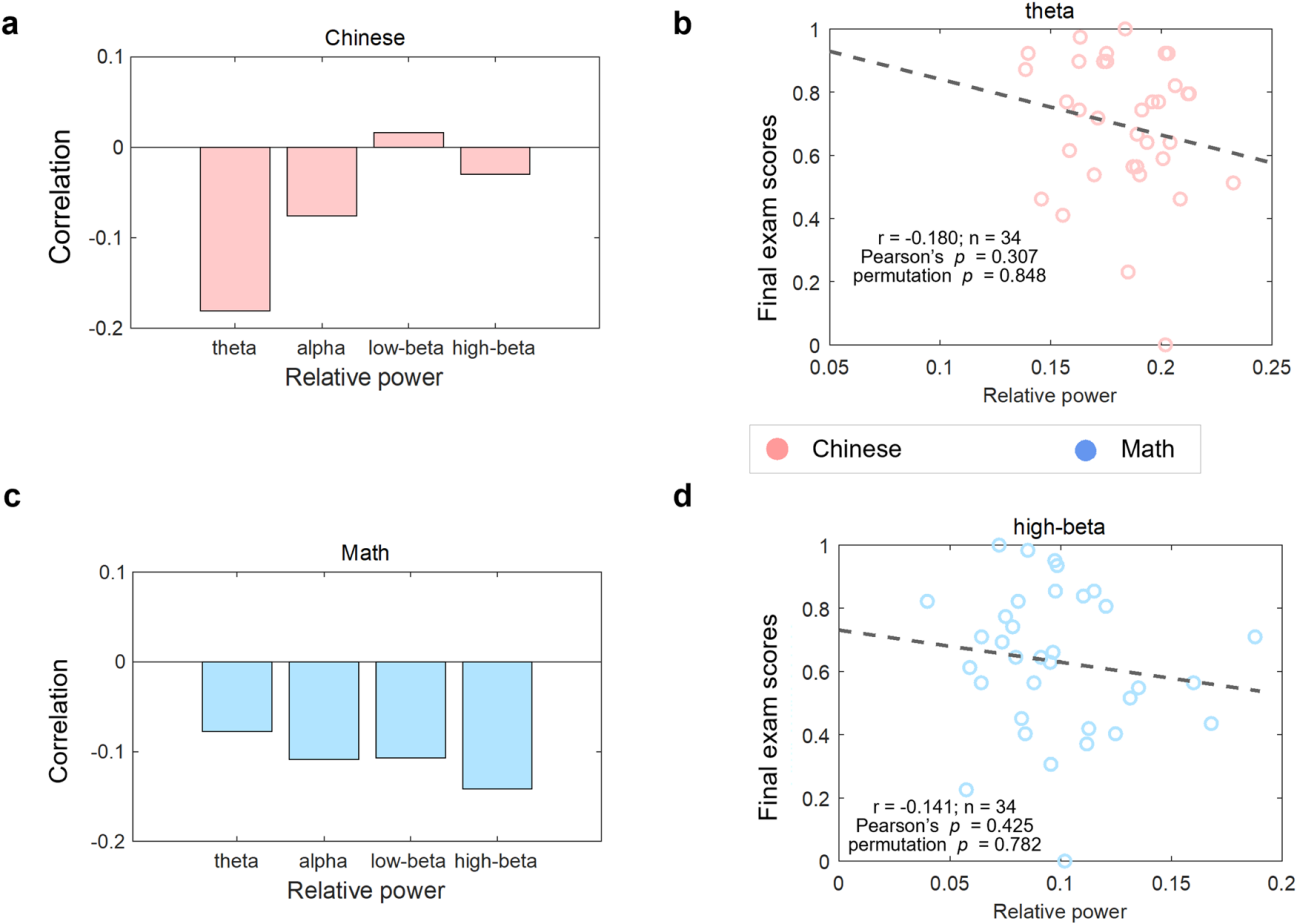
Correlations between single-brain features and learning outcomes. The correlation between the single brain’s relative frequency power in the theta, alpha, low-beta, and high beta band with an individual’s final exam scores of Chinese (**a**) and Math (**c**). No significance was found in any of the frequency bands. **b** Scatter plots between relative power in the theta band and the final exam scores of Chinese (the highest correlation for Chinese). **d** Scatter plots between the relative power in the high-beta band and the final exam scores of Math (the highest correlation for Math).

Additionally, we conducted similar correlational analyses between single-brain EEG features and the final exam scores to test whether single-brain features could also evaluate the learning process. The relative power of the four frequency bands from each student was taken as the single-brain EEG features. As shown in Fig. 7, no significant correlations with the disciplinary final exam scores were found here (Fig. 7b; the highest correlation for Chinese in the theta band: *r* = −0.180, Pearson’s *p* = 0.307, permutation *p* = 0.848, *n* = 34; Fig. 7d; the highest correlation for Math in the high-beta band: *r* = −0.141, Pearson’s *p* = 0.425, permutation *p* = 0.782, *n* = 34).

## Discussion

The learning processes of grade 10 students while taking a soft (Chinese) and a hard (Math) discipline in their real classroom were recorded by wearable EEG devices for a full semester. By using their final exam scores to measure learning outcomes, students with higher Chinese scores were seen to have stronger inter-brain couplings to the top students during the Chinese classes, whereas students with higher Math scores were seen to have stronger inter-brain couplings to other classmates during both the Chinese and the Math courses. Moreover, the inter-brain couplings showed different dominant frequencies for the two disciplines. While the outcome-related inter-brain coupling for Math was found in the theta-band, the importance of the alpha-band was highlighted in Chinese. Our results demonstrate the feasibility of inter-brain coupling to evaluate students’ learning processes for both soft and hard disciplines. More importantly, the present study provides insights into understanding disciplinary differences ‘in the wild’ from an inter-brain perspective.

The correlation between individuals’ student-class coupling and their learning outcomes for Math verified and extended previous findings of neural mechanisms underlying the learning process. By investigating hard disciplines such as physics, biology, and computer science, recent studies have demonstrated student-class coupling as a useful tool to evaluate the learning process^18,19,32^. Our results for Math, another hard discipline, are in line with these studies, where student-class coupling was also found to be correlated with students’ learning outcomes. Moreover, after decomposing data into different frequency bands, our results extended previous findings by showing the importance of the frontal theta-band activity during real-classroom learning. Frontal theta activity has been reported to reflect cognitive processes such as cognitive control^33^, sustained attention^34^, and working memory^35^, and has been found to increase in arithmetic-related tasks^36^. During the learning of hard disciplines, the emphasis on the development of a capacity to master and apply the accepted scientific viewpoints would require the students to align with the course material^6^. Hereby, the theta-band brain activity shared across classmates could reflect students’ continuous engagement with the course content. Then, theta-band student-class coupling could imply the extent to which each student attended the course content^4,19^, or the extent to which each student interpreted the course content^18^. Therefore, better Math learning outcomes are associated with stronger inter-brain couplings to other classmates in the theta band.

The positive correlation between alpha-band student-top coupling during the Chinese sessions and the students’ Chinese final exam scores provides evidence of the critical neural correlates of successful learning in soft disciplines. The distinct frequency band (alpha) compared to Math (theta) suggests that successfully learning Chinese and Math relies on substantially different cognitive processes. Despite the lack of evidence from the neuroscience field on soft-discipline learning, the frontal alpha-band activity could be related to the inhibition of stimulus-driven attention^34,37^ and was involved in tasks with high internal processing demands such as creative ideation^38,39^ and imagination^40^. At the same time, student-top coupling rather than student-class coupling was reflective of the learning outcomes in Chinese, highlighting the neural activities of top students as exemplars for successful learning in soft disciplines. Although the top students might have different internal interpretations of the course content, they could share similar temporal dynamics of the interpretation process. For instance, while learning an ancient poem, two top students could immerse in the aesthetic experience simultaneously when imagining different scenarios in their minds. Note that EEG recording techniques used in our study are beneficial for capturing the temporal dynamics of the learning process rather than the fine-grained representation of the learning content. Taken together, it is reasonable to assume that the temporal dynamics of the frontal alpha activity shared across top students might represent an internal processing state for interpretation construction based on the course content, which is critical for learning Chinese. Moreover, unlike the responses to external stimuli (course content), this internal state may not necessarily be shared across classmates, which may have led to positive results of student-top couplings rather than student-class couplings.

It should also be noted that the non-significant correlation between student-top couplings and the learning outcomes for Math does not necessarily undermine the potential importance of top students for successful Math learning. On the one hand, the correlation coefficients between the Math-session student-top couplings and the Math final exam scores still reached a positive value of >0.2 at the alpha band (Fig. 4d). On the other hand, it may be that the course content was not challenging enough for the top-performing Math students, since classroom teaching was designed to meet the needs of the majority of the class^41–43^. Consequently, top-performing Math students may lose interest in the lecture material and be less inclined to focus on lectures^44^, thereby eliminating any correlations that might have been observed otherwise. By contrast, teaching soft disciplines such as Chinese emphasizes constructive and interpretative activity, which is expected to be similarly challenging for students at different proficiency levels.

By computing the correlation between theta-band student-class couplings during the Chinese sessions and learning outcomes for Math, we explored whether our findings on theta-band student-class couplings were discipline-specific. As the theta-band student-class couplings during both Chinese and Math sessions were found to be associated with the learning outcomes for Math, this observation might imply a discipline-general role of the theta-band student-class couplings. Although previous studies have mainly focused on one discipline per study, student-class coupling has been reported to be able to characterize students’ learning processes in different disciplines, including biology, chemistry, and computer sciences^4,18,19^. Together with our theta-band results, it would be reasonable to consider the theta-band student-class couplings in the present study could reflect the students’ capabilities (cognitive control, sustained attention, working memory, etc.) to control themselves and engage in the course content that could support discipline-general learning. The theta-band student-class couplings in Chinese sessions could reflect the students’ general capabilities and therefore be informative of their learning outcomes for Math, a typical hard discipline^6^. Nevertheless, the non-significant correlation between theta-band student-class coupling (during both the Chinese and Math sessions) and the Chinese final exam scores might suggest a relatively loose link between students’ capabilities supporting the course content engagement and the Chinese learning outcomes due to the nature of the soft discipline^7,10^. This piece of result echoed a previous study reporting a more important contribution of the students’ studiousness and continuous engagement in learning Math than German^45^. Notably, no significant correlation was found between the student-“Chinese-top” couplings during the Math sessions and the learning outcomes for Chinese and vice versa. It was possible that the top-performing students mainly exhibited discipline-specific optimal learning processes only in their corresponding disciplinary courses. In other words, the top-performing students might serve as a discipline-specific reference to represent effective learning in their corresponding disciplines rather than a general reference across disciplines. Therefore, the student-top couplings in the present study were only related to discipline-specific learning outcomes. Together, these results provide more insights into the discipline specificity of the inter-brain couplings.

In the present study, the “hard-soft” dimension was used to investigate how disciplinary differences manifested in the classroom learning process from a neuroscience perspective. While disciplinary differences were observed as reflected by distinct inter-brain coupling patterns, our findings also indicated that some cognitive processes might be shared across the learning process of these two disciplines. Therefore, in addition to Chinese and Math, more disciplines would be needed to clarify the discipline-specific and discipline-general process thoroughly during real-world classroom learning^46^. Moreover, considering that the “hard-soft” dimension is contentious^47^, a deeper understanding of the commonalities and differences among disciplines from a neuroscience perspective might also provide insight to help us re-consider the framework of disciplinary differences.

Several limitations must be noted in the present study investigating the disciplinary differences in students’ successful learning process in real-classroom settings. First, the present ecologically valid paradigm posed a challenge to strictly-controlled contrasts between disciplines. Multiple factors (e.g., learning contents, learning goals, and learning difficulties) could lead to distinct inter-brain coupling patterns in soft and hard disciplines. While this is how disciplinary differences manifest in everyday learning processes, future work will be needed to clarify the unique contributions of these factors. Second, while the present effect sizes in the range of 0.1 to 0.2 (the correlation between inter-brain couplings and learning outcomes for Chinese: *r*^2^ = 0.187; for Math: *r*^2^ = 0.115) are in general comparable to neuroscience studies in a variety of fields (e.g., perceptual decision-making^48^, working memory^49^, social interaction^50^, etc.), they were relatively small as compared to recent inter-brain studies on learning that have reported *r*^2^ values varying from 0.1 to 0.6. The relatively small effect sizes in the present study could be attributed to a number of factors, such as the noisy real-classroom environment, the limited recording channels, and frontal coverage, etc. The development of portable EEGs with larger coverage areas or portable functional near-infrared spectroscopy (fNIRS) devices^51^ to support daily longitudinal real-classroom recordings in the near future. Third, the reported significant correlational results were based on uncorrected *p* values. Although a multiple comparison correction considering the number of frequency bands, the number of top students, as well as the two disciplines would make these results less significant, the results were not likely to be spurious correlations: (1) The correlations between alpha-band student-top couplings and Chinese final exam scores remained significant with the number of top students varying from 2 to 6, suggesting a robust phenomenon. Specifically, performing an FDR (False Discovery Rate) correction^52^ in the condition with the strongest correlation (top students’ number = 4, Fig. 4b) yielded a corrected significant *p* value of 0.04 with both the disciplines (2, Chinese and Math) and frequency bands (4, theta to high-beta) considered. While the results could be insignificant when taking the number of top students into consideration, such a practice could be too strict and further method development with an idea similar to the well-established cluster-based permutation tests widely applied in the field of neuroscience for identifying robust significant results are expected^53^; (2) In addition, the comparison between the obtained correlation coefficient and the corresponding permuted null distribution could provide another indirect support for the validity of the Math result. Specifically, the absolute difference (*r* difference = 0.341) between the true correlation coefficient (*r* = 0.339) and the mean value of the null distribution (*r* = −0.002) is comparable to the Chinese results (*r* differences ranged from 0.309 to 0.388 with the varying number of top students). It should also be noted that, whereas the mean value of the null distribution was around zero for the correlations between theta-band student-class couplings and Math final exam scores, the mean values were well above zero (e.g., ranging from 0.132 to 0.217 for the theta-band correlations between student-top couplings and Math scores, Fig. 3d), providing another evidence for the effectiveness of the nonparametric permutation tests. Therefore, the moderately-significant correlations are believed to reflect non-random, reliable findings.

In conclusion, the present study investigated the disciplinary differences in students’ real-world classroom learning by recording their brain signals with wearable EEG devices. Our results demonstrated the potential of using inter-brain coupling to evaluate students’ learning processes for both soft and hard disciplines. More importantly, the present study provides empirical evidence of disciplinary differences from a neuroscience perspective, advancing our understanding of how disciplinary differences manifest in the everyday learning.

## Methods

### Participants

Thirty-six grade 10 students (sex: 16 females; age: 15–16 years old) from the same class (37 students in total. One student chose not to participate in the present study) from a high school in Beijing wore a headband EEG device during their regular Math and Chinese lessons for one semester. Two students were omitted from the analysis due to the consistently poor contact of EEG Fp1 electrodes throughout recordings (>85% of epochs were removed, see more details in the “Data preprocessing” section). Therefore, a total of 34 students were included in the classroom learning EEG analysis. Among these students, 22 of them were involved in another task session at the end of the semester, in which they were asked to join an eye-close/open task for EEG signals validation.

The study was conducted in accordance with the Declaration of Helsinki, and the protocol was approved by the ethics committee of the Department of Psychology, Tsinghua University (THU201708). All the participants and their legal guardians gave their written informed consent.

### Procedure and data recording

In the present study, a dual-channel headband with dry electrodes was used to record EEG at Fp1 and Fp2 over the forehead at a sampling rate of 250 Hz (Brainno, SOSO H&C, South Korea). The reference electrode was placed on the right ear lobe with a ground at Fpz. The EEG headbands have previously been used in various tasks, including sudoku games^54^, movie-watching^55^, real-world surgeries^56,57^, and foreign language learning^58^. In these studies, EEG signals recorded by the headband were able to detect concentration state during sudoku games^54^ and classify the “known/unknown word” when reading texts on the screen^58^. The headband was also used in an inter-brain coupling study during movie-watching in which increased inter-brain coupling among participants was observed when emotional-aroused or informative scenes appeared^55^. In addition, this EEG headband was used to monitor the intraoperative real-time stress in degenerative lumbar spine surgery^56^. Different frequency bands of EEG signals covering the theta, alpha, and beta band have been used in these studies. Together, these studies validated the effectiveness of this headband in monitoring EEG signals in a real-life context.

Moreover, the present study conducted an additional eye-closed/open task to further test the signal quality of the headband in the classroom environment. The eye-closed/open task was chosen since the reduction in alpha-band power comparing the eye-closed and eye-open task is a reliable physiological phenomenon observed in most people^59^. Therefore, the eye-closed/open task has been widely used for the data quality validation of EEG devices^60^. Here, students were required to open and close their eyes for 2 min respectively when sitting in their classroom; then, we found a spectral peak in the alpha range in the eye-closed condition compared to the eye-open condition as expected (Supplementary Fig. S1). Previous studies together with the validation task in the present study demonstrated the validity of the EEG headbands.

The data collection lasted for 4 months to cover the whole semester. For each month, students’ EEG signals during Chinese sessions and Math sessions were recorded for 1 week (one session or two sessions per day) following the regular curriculum. The total number of sessions was 39, with 19 sessions for Chinese and 20 sessions for Math. Before Chinese or Math sessions began, students wore headbands with the help of experimenters, and the headbands were taken off after each session. Each session lasted for 40 min. There was one Chinese session where EEG devices failed to record any data due to technical issues. A total of 18 sessions for Chinese and 20 sessions for Math were included in the following analysis.

During the Chinese and Math sessions, the learning content was taught according to the arrangement of the school. The Math sessions include the introduction to planar vectors, cubic geometry, plural, statistics, and probability; the Chinese sessions include reading ancient and modern poems, essays, and novels and the introduction to writing.

Besides wearing the EEG headbands, the students participated in their classes as usual. Their final exam scores in Chinese and Math were taken as indicators of their learning outcomes. No pre-tests were organized before the start of the study. Compared with the specially-designed quizzes usually used in laboratory-based studies, final exams were expected to boost ecological validity as they were derived from the highly-developed evaluation system in daily educational practice. The final exams covered the contents of the whole semester. Both exams were scored out of 100. The median of the students’ Math scores was 73, ranging from 33 to 95, and the median of the students’ Chinese scores was 69, ranging from 40 to 79. The scores were sufficiently diverse to characterize students’ differences in learning outcomes. These scores were normalized to [0, 1] using a min-max transformation for the following analysis.

### Data preprocessing

Since EEG data were recorded in a regular classroom environment and students were instructed to attend Chinese and Math sessions as usual, more artifacts were expected compared to conventional, highly-controlled laboratory settings. In the present study, there were three types of prominent artifacts: (1) a high value indicating signal saturation possibly due to losing contact with the headband; (2) slow drifts related to extensive head or body movements; (3) ocular artifacts related to eye movements.

The recorded EEG data were segmented into non-overlapping 30-s epochs for preprocessing^61,62^. As shown in Supplementary Fig. S2, ratios for saturated samples per epoch illustrated a two-tailed distribution that most epochs containing saturated samples for less than 10% or more than 90%. Therefore, 50% was chosen as a threshold empirically. One epoch would be rejected if it contained saturated samples for more than 50%. The remaining epochs were then processed to remove the slow drifts with the NoiseTools toolbox^63^ under Matlab (MathWorks, USA). The removal of the slow drifts was achieved by using the nt_detrending() function. By estimating the position of the glitch, this function could perform a weighted polynomial fit and achieve a better fit to the non-glitch parts. The processed epochs were further band-pass filtered between 0.1 Hz and 40 Hz with 1-s zero-padding at both ends. Afterward, the ocular artifacts were attenuated with the MSDL (multi-scale dictionary learning) toolbox, which was efficient in ocular artifacts removal for single-channel EEG signals^64^. Epochs were decomposed into neuronal and non-neuronal sources with dictionary learning. Then, the coefficients of non-neuronal sources were set to zero to achieve artifact reduction with the seq_MSDL() function. Supplementary Figs. S3 and S4 illustrated representative examples before and after the artifacts rejection procedure. Finally, epochs were rejected automatically if any samples in any channels exceeded a $\pm$150 μV threshold. With the above preprocessing procedure, 57.2 ± 1.85% epochs were retained per student, ranging from 31.2 to 76.7%. The data retention rate was comparable with previous EEG studies in classroom settings^4,17^. The number of retained epochs per session per student was shown in Supplementary Fig. S5.

### Data processing

The total interdependence (TI) method has been employed in the present study to calculate the inter-brain coupling^4,17,31^. Recent inter-brain studies have validated the efficiency of TI methods in tracking individuals’ engagement and valence levels in naturalistic scenarios such as a classroom and a concert hall^4,65^. TI was estimated by computing the magnitude squared coherence using the Welch method when clean 30-s epochs were available at the same moments from both students. For ${X}_{i}$, a 30-s epoch from a certain student $i$ and ${X}_{j}$, an overlapping epoch from another student $j$, TI value was calculated as follows:

1 $${{\rm{TI}}}_{{X}_{i},{X}_{j}}=-\frac{2}{{f}_{s}}\mathop{\sum }\limits_{m=1}^{M}{\mathrm{ln}}(1-{C}_{{X}_{i},{X}_{j}}^{2}\left(m\triangle f\right))\triangle f$$

2 $$\triangle f=\frac{{f}_{s}}{2(M-1)}$$

Here, ${C}_{{X}_{i},{X}_{j}}()$ is the magnitude squared coherence calculation, ${f}_{s}$ is the sampling rate, *M* is the number of desired frequency points in the interval between 0 and the Nyquist frequency $\frac{{f}_{s}}{2}$. The $\triangle f$ is the frequency resolution. Theta, alpha, low-beta and high-beta TI were computed by summing the coherence values within 4–8 Hz, 8–13 Hz, 13–18 Hz, and 18–30 Hz, respectively.

TI for one pair of students for each session was obtained by averaging TI values across all epochs and the two recording electrodes. A minimum of six artifact-free common epochs for paired students were included for further analysis. The lower limit was empirically chosen to get a comparable minimum data amount for each pair of students with the previous studies^4,17^. In total, 96.6% of TI values for each pair and each epoch remained for the following analysis. A $N* N$ pairwise TI matrix ($N$ is the number of students) could be obtained for each session. TI values within the matrix were then normalized to [0,1] for each session, following the practice in previous studies^4,17^. Then, the matrixes were averaged across $K$ sessions to obtain an averaged inter-brain coupling for each pair of students for a specific discipline ($K=18$ for Chinese and $K=20$ for Math).

Then, student-class coupling for student $i$ was obtained by averaging TI values over all possible pairwise combinations between the student $i$ and the rest of the class. Student-top coupling for student $i$ was computed by averaging TI values over all possible pairwise combinations between the student $i$ and the top students except themselves if included. Therefore, for each student, there would be a student-class coupling value and a student-top coupling value as indicators of disciplinary learning process for the whole semester for each frequency band. Please refer to Fig. 2 for the schematic illustration of the inter-brain coupling analysis.

Furthermore, Pearson’s correlations between individuals’ student-class coupling (or student-top coupling) and their corresponding learning outcomes were calculated for soft and hard disciplines separately. For student-class coupling, all students were included in the correlation analysis. For student-top coupling, however, the top students themselves were not included during the correlation analysis between student-top couplings and learning outcomes. For example, if individuals’ student-top coupling was computed with the top ${N}_{E}$ students, then the top ${N}_{E}$ students’ coupling values, as well as their learning outcomes, would be removed, leaving $(N-{N}_{E})$ out of the $N$ students for conducting correlations. Note that the top students included in this analysis differed in different disciplines. For the correlation analysis between student-top couplings during Chinese sessions and learning outcomes for Chinese, the top students are students who had top performances in Chinese and vice versa. The effect of top students’ number on the relationship between student-top couplings and learning outcomes was analyzed to evaluate the reliability of possible findings.

Then, a further exploratory analysis was conducted to test whether the possibly-existing correlations between the inter-brain couplings and the corresponding disciplinary learning outcomes are discipline-specific. Specifically, as discussed in the Introduction section, top-performing students in a specific discipline may serve as an exemplar for the optimal disciplinary learning process. Therefore, for possibly-existing results in the student-top couplings, we explored whether the role of exemplar for top-performing students was discipline-specific by re-computing the correlation between student-“Chinese-top” coupling during Math sessions and the final exam scores of Chinese and vice versa. For student-class coupling, as the ‘class’ (i.e., all other classmates) are the same for Math and Chinese sessions, the discipline-specificity of student-class coupling was investigated by switching the disciplinary scores (i.e., computing the correlation between student-class couplings during Math sessions and the final exam scores of Chinese and vice versa).

Additionally, we conducted similar correlational analyses between single-brain EEG features and the final exam scores for comparison. The relative power of each frequency band of interest (theta, alpha, low-beta and high-beta) was obtained by dividing the power in the 1–40 Hz band after a fast Fourier transform for each 30-s epoch. Then, values of the relative power of each frequency band of interest were averaged across all epochs and all sessions within each discipline for each student as the single-brain EEG features. Finally, Pearson’s correlations between individuals’ single-brain EEG features and corresponding learning outcomes were calculated separately for soft and hard disciplines.

### Nonparametric permutation tests

We conducted nonparametric permutation tests to further verify the correlation between inter-brain coupling and learning outcomes. In the permutation for student-top coupling, we randomly selected N students out of all the students in the class rather than the real top N students. The student-“top” coupling was re-calculated according to this new selection, and Pearson’s correlation analyses between the student-“top” coupling and learning outcomes in Chinese were re-conducted. A null distribution of the correlation between student-top couplings and learning outcomes was yielded by 5000 permutations. The statistical significance of the original correlation was assessed by comparing it with values generated by the 5000 shuffled versions. For student-class coupling, however, since there is only one way to select the reference students (i.e., all the other students), the final-exam scores for each student were randomly disrupted and re-assigned to students in the permutation. We then re-analyzed the correlation between student-class coupling and disrupted scores. The statistical significance was also assessed by comparing the original correlation values with those values generated by the 5000 shuffled versions. The parametric and nonparametric tests together are expected to provide a more comprehensive assessment of the results^66,67^: correlations were regarded as significant only if both Pearson’s *p* and permutation *p* were smaller than 0.05.

### Reporting summary

Further information on research design is available in the Nature Research Reporting Summary linked to this article.

## Supplementary information

Supplementary Materials

Reporting Summary

## Data Availability

The data supporting this work can be found in https://cloud.tsinghua.edu.cn/d/455601fcaf814baca98d/.

## Code availability

The codes supporting this work can be found in https://cloud.tsinghua.edu.cn/d/455601fcaf814baca98d/.
