# Supplementary material for: Inter-brain coupling reflects disciplinary differences in real-world classroom learning
Source: NPJ Sci Learn. 2023 May 2;8:11. doi: 10.1038/s41539-023-00162-1 (PMC10154329; doi:10.1038/s41539-023-00162-1)
Supplement: Supplementary file 1 — Supplementary Materials [file 41539_2023_162_MOESM1_ESM.pdf]

Supplementary Materials for

Inter-brain coupling reflects disciplinary differences in real-world classroom learning

Jingjing Chen, Penghao Qian, Xinqiao Gao, Baosong Li, Yu Zhang & Dan Zhang

*Corresponding author.
Dan Zhang, dzhang@tsinghua.edu.cn, +86-10-62796737
Yu Zhang, zhangyu2011@tsinghua.edu.cn, +86-10-62785686

This PDF file includes:

Table S1

Figs. S1 to S5

Table S1. Statistics (Pearson's *p* values and permutation *p* values) in the correlations between inter-brain coupling and learning outcomes.

Chinese				
	**theta**	**alpha**	**Low-beta**	**High beta**
**Top 2 students**	0.202/0.163	**0.036/0.016**	0.263/0.046	0.645/0.489
**Top 3 students**	0.230/0.199	**0.024/0.010**	0.444/0.067	0.538//0.436
**Top 4 students**	0.347/0.279	**0.017/0.005**	0.612/0.096	0.415/0.364
**Top 5 students**	0.077/0.060	**0.030/0.010**	0.900/0.185	0.934/0.746
**Top 6 students**	0.186/0.148	0.061/0.020	0.754/0.105	0.932/0.768
**All other classmates**	0.534/0.258	0.665/0.322	0.347/0.824	0.420/0.204
**Math**				
	**theta**	**alpha**	**Low-beta**	**High beta**
**Top 2 students**	0.283/0.376	0.182/0.192	0.070/0.049	0.229/0.168
**Top 3 students**	0.861/0.791	0.102/0.125	0.056/0.040	0.854/0.560
**Top 4 students**	0.652/0.735	0.156/0.186	0.118/0.094	0.669/0.811
**Top 5 students**	0.795/0.824	0.075/0.087	0.448/0.367	0.533/0.877
**Top 6 students**	0.556/0.741	0.194/0.249	0.825/0.643	0.409/0.923
**All other classmates**	**0.0498/0.024**	0.270/0.130	0.512/0.253	0.578/0.281

Note. Correlations were regarded as significant only if both Pearson's *p* and permutation *p* are smaller than 0.05. All the *p* values were uncorrected.

Fig. S1.

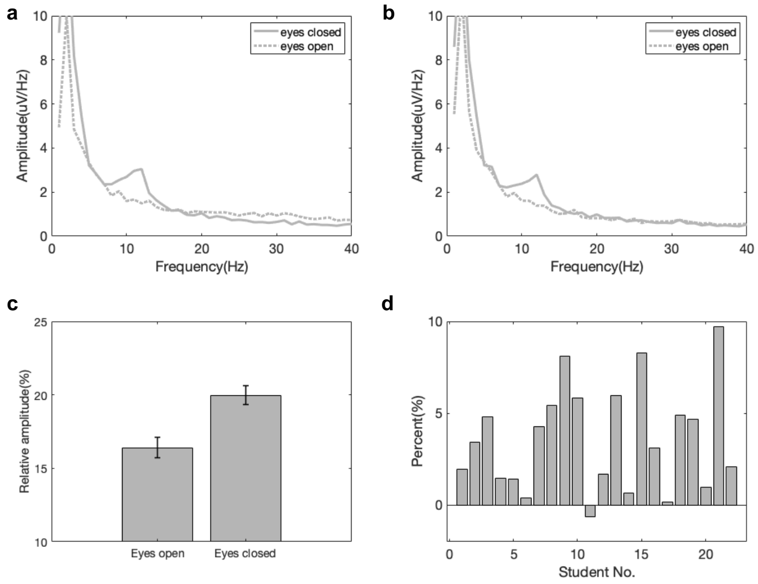

The signal quality validation of EEG headbands in a 2-minute eye-closed/open task. Twenty-two students out of the same class volunteered to participate in this task. Students were required to open and close eyes for two minutes respectively when sitting in their classroom. Then, fast Fourier transform was conducted to compare the frequency spectral characteristics of EEG signals between conditions. **a,** The frequency spectra for a representative student at Fp1. The solid line represents the eye-closed condition, and the dashed lines represent the eye-open condition. **b,** The frequency spectra for the same student at Fp2. **c,** Relative amplitudes of the alpha band (8-13 Hz) in the eye-open and eye-closed conditions for all the students. Errorbar indicates standard deviation. The amplitudes were averaged across Fp1 and Fp2; **d,** Percent of relative amplitude differences (eye-closed minus eye-open) at the alpha band for each student. The amplitudes were averaged across Fp1 and Fp2.

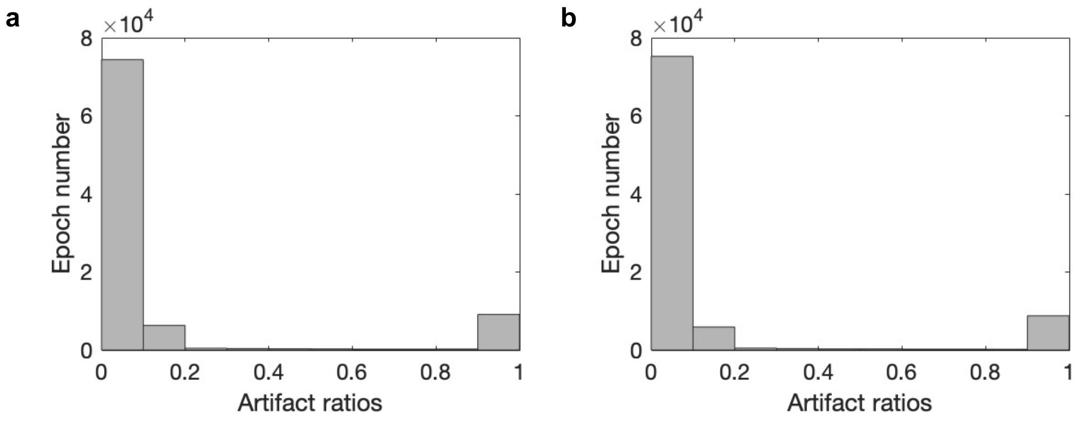

Fig. S2: The distribution of the artifacts ratios per epoch for (**a**) Fp1 and (**b**) Fp2. The ratios for saturated samples per epoch illustrated a 2-tailed distribution.

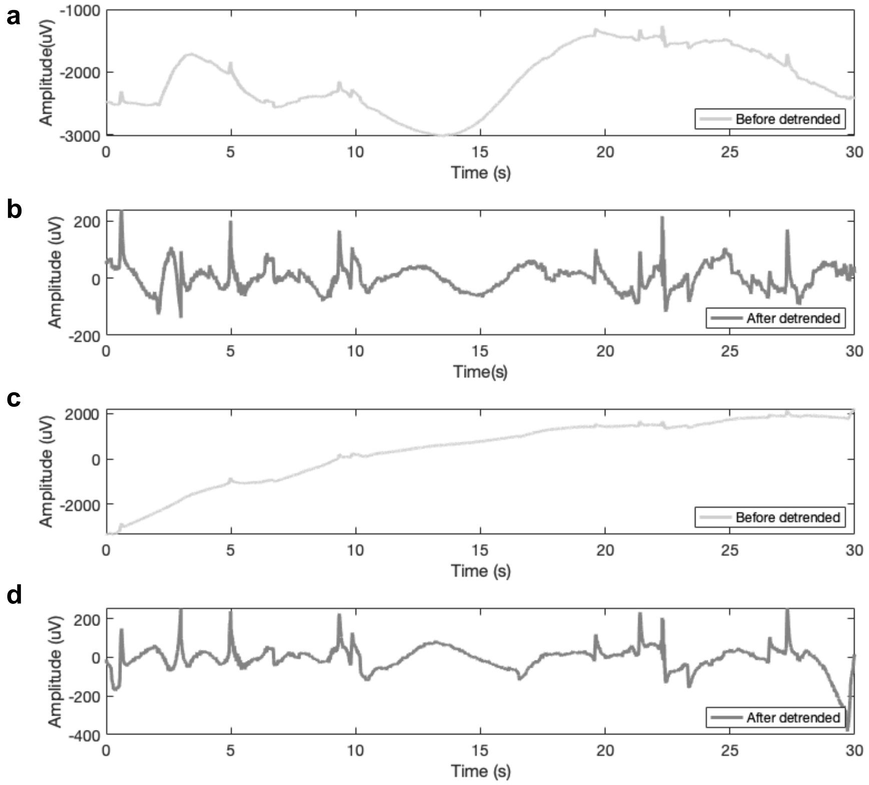

Fig. S3: An illustration of a representative EEG epoch before and after the detrending procedure with the Noisetool toolbox (**a, b**) at Fp1; (**c, d**) at Fp2. The lines with a lighter color indicated the condition before detrending. The lines with a darker color indicated the condition after detrending. The slow drift was removed after the detrending procedure.

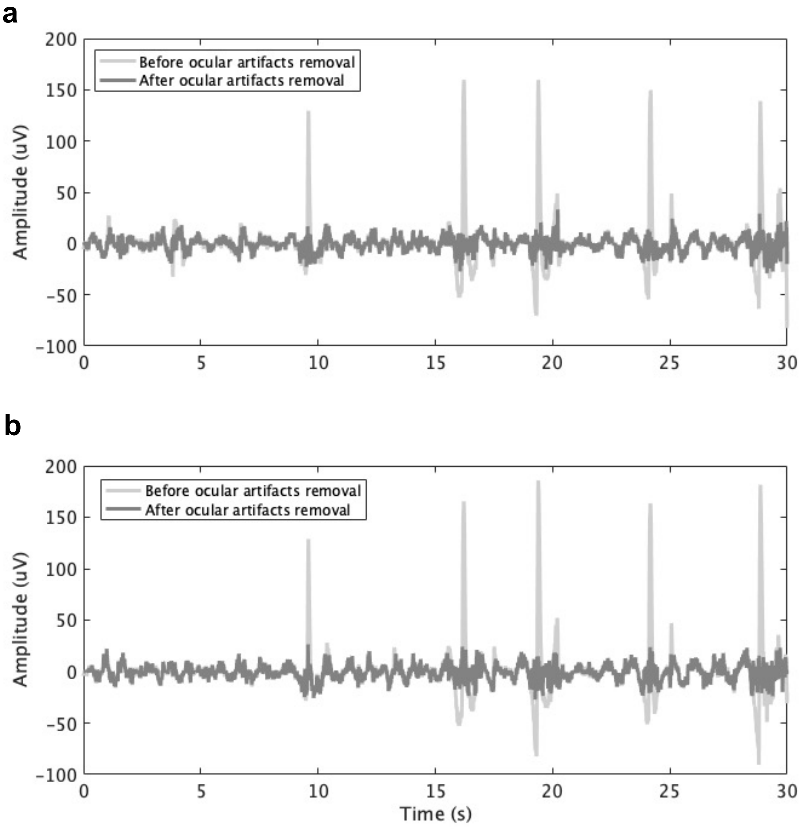

Fig. S4: An illustration of a representative EEG epoch before and after the ocular artifact removal procedure with the MSDL toolbox (**a**) at Fp1; (**b**) at Fp2. The lines with a lighter color indicated the condition before ocular artifact removal. The lines with a darker color indicated the condition after ocular artifact removal. The ocular artifact was removed after the procedure.

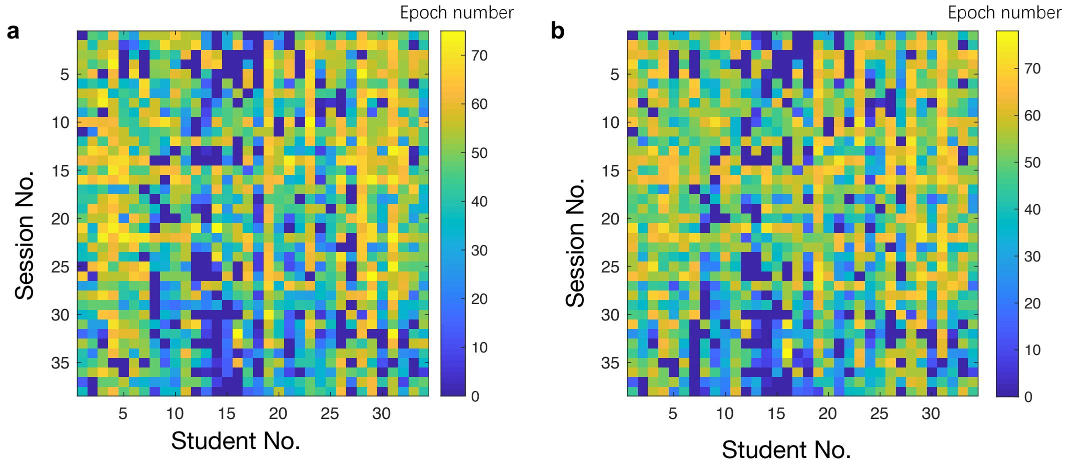

Fig. S5: The number of retained epochs per session per student (**a**) at Fp1; (**b**) at Fp2 after the preprocessing procedure. The colorbar indicated the number of retained epochs.
