# Supplementary material for: Inter-brain coupling reflects disciplinary differences in real-world classroom learning
Source: NPJ Sci Learn. 2023 May 2;8:11. doi: 10.1038/s41539-023-00162-1 (PMC10154329; doi:10.1038/s41539-023-00162-1)
Supplement: Supplementary file 2 — Reporting Summary [file 41539_2023_162_MOESM2_ESM.pdf]

nature portfolio

Corresponding author(s): Dan Zhang

Last updated by author(s): Sep 10, 2022

Reporting Summary

Nature Portfolio wishes to improve the reproducibility of the work that we publish. This form provides structure for consistency and transparency in reporting. For further information on Nature Portfolio policies, see our Editorial Policies and the Editorial Policy Checklist.

Statistics

For all statistical analyses, confirm that the following items are present in the figure legend, table legend, main text, or Methods section.

n/a	Confirmed	
☐	☒	The exact sample size (n) for each experimental group/condition, given as a discrete number and unit of measurement
☐	☒	A statement on whether measurements were taken from distinct samples or whether the same sample was measured repeatedly
☐	☒	The statistical test(s) used AND whether they are one- or two-sided<br>*Only common tests should be described solely by name; describe more complex techniques in the Methods section.*
☒	☐	A description of all covariates tested
☐	☒	A description of any assumptions or corrections, such as tests of normality and adjustment for multiple comparisons
☐	☒	A full description of the statistical parameters including central tendency (e.g. means) or other basic estimates (e.g. regression coefficient) AND variation (e.g. standard deviation) or associated estimates of uncertainty (e.g. confidence intervals)
☐	☒	For null hypothesis testing, the test statistic (e.g. F, t, r) with confidence intervals, effect sizes, degrees of freedom and P value noted<br>*Give P values as exact values whenever suitable.*
☒	☐	For Bayesian analysis, information on the choice of priors and Markov chain Monte Carlo settings
☒	☐	For hierarchical and complex designs, identification of the appropriate level for tests and full reporting of outcomes
☐	☒	Estimates of effect sizes (e.g. Cohen's d, Pearson's r), indicating how they were calculated

Our web collection on statistics for biologists contains articles on many of the points above.

Software and code

Policy information about availability of computer code

Data collection	N/A
Data analysis	The analysis were conducted based on the standard function and custom codes in Matlab.The codes supporting this work can be found in https://cloud.tsinghua.edu.cn/d/455601fcaf814baca98d/.

For manuscripts utilizing custom algorithms or software that are central to the research but not yet described in published literature, software must be made available to editors and reviewers. We strongly encourage code deposition in a community repository (e.g. GitHub). See the Nature Portfolio guidelines for submitting code & software for further information.

Data

Policy information about availability of data

All manuscripts must include a data availability statement. This statement should provide the following information, where applicable:

- Accession codes, unique identifiers, or web links for publicly available datasets
- A description of any restrictions on data availability
- For clinical datasets or third party data, please ensure that the statement adheres to our policy

The data supporting this work can be found in https://cloud.tsinghua.edu.cn/d/455601fcaf814baca98d/.

Human research participants

Policy information about studies involving human research participants and Sex and Gender in Research.

Reporting on sex and gender	The biological sex information was collected based on the self-reporting in the information consent. Recruitment and analysis did not differ by sex. The ratio of sex for the participants are not pre-defined but follow the ratio of the real-world class in the high school. No further sex-based analyses were conducted.
Population characteristics	Thirty-six students (sex: 16 females; age: 15-16 years old) from the same class in grade 10 of a high school in Beijing participated in the present study. All students are Chinese.
Recruitment	The recruitment was conducted by contacting high schools in Beijing. The results of recruitment was solely decided by the willingness of schools, teachers, students and their legal guardians since it is challenging to to get permission to record EEG signals during the real-world classroom learning for a whole term.
Ethics oversight	The study was conducted in accordance with the Declaration of Helsinki, and the protocol was approved by the ethics committee of the Department of Psychology, Tsinghua University (THU201708).

Note that full information on the approval of the study protocol must also be provided in the manuscript.

Field-specific reporting

Please select the one below that is the best fit for your research. If you are not sure, read the appropriate sections before making your selection.

☐ Life sciences ☒ Behavioural & social sciences ☐ Ecological, evolutionary & environmental sciences

For a reference copy of the document with all sections, see nature.com/documents/nr-reporting-summary-flat.pdf

Behavioural & social sciences study design

All studies must disclose on these points even when the disclosure is negative.

Study description	Quantitative, EEG and behavior
Research sample	Thirty-six students (16 females; age: 15-16 years old) from the same class in grade 10 of a high school in Beijing participated in the present study. All students are Chinese.
Sampling strategy	The sampling size and sampling population was solely driven by the willingness of schools, teachers, students and their legal guardians. No statistical methods were used to predetermined sample size, but the present sample is comparable with previous real-world classroom EEG studies such as Dikker, 2017 and Bevilacqua, 2019.
Data collection	In the present study, a dual-channel headband with dry electrodes was used to record EEG at Fp1 and Fp2 over the forehead at a sampling rate of 250 Hz (Brainno, SOSO H&C, Korea). The reference electrode was placed on the right ear lobe with a ground at Fpz. The data collection lasted for four months to cover the whole semester. For each month, students' EEG signals during Chinese sessions and Math sessions were recorded for one week (one session or two sessions per day) following the regular curriculum. The final exam scores for students were collected as indicators of the learning outcomes.
Timing	The data were collected in the spring term of 2018 acadamic year.
Data exclusions	There was one Chinese session when EEG devices failed to record any data due to technical issues. Two students were omitted from the analysis due to the consistently poor contact of EEG Fp1 electrode across sessions (>85% of epochs were removed, see more details in the data preprocessing section).
Non-participation	No participants dropped out from the EEG recordings during real-world classroom learning.
Randomization	All comparisons described in the manuscript were conducted within subjects.

Reporting for specific materials, systems and methods

We require information from authors about some types of materials, experimental systems and methods used in many studies. Here, indicate whether each material, system or method listed is relevant to your study. If you are not sure if a list item applies to your research, read the appropriate section before selecting a response.

Materials & experimental systems

n/a	Involved in the study
☒	☐ Antibodies
☒	☐ Eukaryotic cell lines
☒	☐ Palaeontology and archaeology
☒	☐ Animals and other organisms
☒	☐ Clinical data
☒	☐ Dual use research of concern

Methods

n/a	Involved in the study
☒	☐ ChIP-seq
☒	☐ Flow cytometry
☒	☐ MRI-based neuroimaging
